# Exploration vs. Data Refinement via Multiple Mobile Sensors

**DOI:** 10.3390/e21060568

**Published:** 2019-06-05

**Authors:** Mohammad Shekaramiz, Todd K. Moon, Jacob H. Gunther

**Affiliations:** Electrical and Computer Engineering Department and Information Dynamics Laboratory, Utah State University, 4120 Old Main Hill, Logan, UT 84322-4120, USA

**Keywords:** sensor configuration, adaptive sampling, exploration, data refinement, mobile sensors, epistemic utility controller, Gaussian process regression (GPR), decision under conflict

## Abstract

We examine the deployment of multiple mobile sensors to explore an unknown region to map regions containing concentration of a physical quantity such as heat, electron density, and so on. The exploration trades off between two desiderata: to continue taking data in a region known to contain the quantity of interest with the intent of refining the measurements vs. taking data in unobserved areas to attempt to discover new regions where the quantity may exist. Making reasonable and practical decisions to simultaneously fulfill both goals of exploration and data refinement seem to be hard and contradictory. For this purpose, we propose a general framework that makes value-laden decisions for the trajectory of mobile sensors. The framework employs a Gaussian process regression model to predict the distribution of the physical quantity of interest at unseen locations. Then, the decision-making on the trajectories of sensors is performed using an epistemic utility controller. An example is provided to illustrate the merit and applicability of the proposed framework.

## 1. Introduction

We consider the general problem of exploration using multiple mobile sensors, or fixed sensors whose field of view is reconfigurable. The problem is to *locate* regions where concentration of the physical quantity of interest occurs and then, having found such regions, to expend sensor capability to *refine* data there while continuing to search for new interesting regions. That is, after initial discovery, there is a trade-off between increasing knowledge by taking more measurements in regions already known to be of interest and increasing knowledge by exploring in regions where concentration of the phenomenon of interest (PoI) may exist but are not known to be present. For the purpose of brevity, we refer to regions containing a high concentration of the PoI as *interesting regions*. Due to the uncertainties of exploration, the problem is not posed as one of optimal path (or resource) planning, but as a problem that balances the competing imperatives of refining measurements while exploring new territory. Therefore, the problem studied here is different than the problems raised in other research fields such as exploration and path planning in robotics and unmanned aerial vehicles (UAVs) [1,2,3,4,5,6,7,8].

There exist various approaches for sensor array configuration or sensor placement [9,10,11,12,13,14,15,16,17,18]. For example, Zhang provides a necessary condition for optimal sensor placement in two-dimensional space using the algebraic structure of sensors [10]. The problem of sensor array configuration in a remote sensing formulation is addressed in [19], in which a statistical optimal criterion is used to identify a solution. By contrast, in this paper, a more dynamic, exploratory stance is taken to trade off typically repeated (or related) measurements against measurements in new areas. Here, we focus on the Gaussian processes (GPs) modeling to formulate the sensor placement problem [13,14,15,16,17,18]. A Gaussian process, as a Bayesian nonparametric tool, is useful for modeling spatiotemporal data, especially in cases where the data contain random variations and so it cannot be well-represented via parametric models [20]. GPs have been used for decades as a supervised learning tool for regression problems known as Gaussian process regression (GPR) models [21,22], and are also referred to as *kriging*, named after the mining engineer D.G. Krige in the geostatistics literature [23,24,25]. GPR models are used here as a spatiotemporal interpolator/extrapolator tool to predict the PoI at unsampled data points. GPR models and kriging methods are applicable to a wide variety of problems such as modeling or control of robotics-related applications, the prediction and estimation of temperature, precipitation, missing pixels and unmixing of pixels in hyperspectral imaging (HSI), human head pose estimation, concentration of carbon dioxide in the atmosphere, etc. [22,26,27,28,29,30,31,32,33,34,35]. As an example in HSI, one main objective is to unmix the spectral information to make an inference of the composing materials in the scene. Imbiriba et al. in [30] consider a nonlinear model where the underlying function is governed by a Gaussian process model to detect the nonlinearly mixed pixels. Xing et al. in [33] introduce an algorithm for dictionary learning based on a GP prior to remove the noise and infer the missing data in HSI. Another example is to predict the temperature based on the available data collected from the meteorological stations. For instance, Wu and Li [31] apply the residual kriging method to predict the average monthly temperature at 500 unknown locations in the United States.

More closely related to this research, GPs have also found been applied in sensor placement problems [13,14,15,16,17,18]. In [15], Garnett et al. propose a Bayesian optimization algorithm for sensor placement based on GP modeling. They applied their approach to place 50 sensors around the UK to measure the air temperature. A typical sensor placement technique is to use the variances associated with the maximum a posteriori (MAP) estimates of GP as a measure representing the amount of uncertainty in the region. This leads to placing the sensors at locations with the highest variance (entropy) [17,18], in order to reduce the overall entropy in the region under study. This characterization of the quality of sensor placements seems to be naive due to the following reasons. As observed by [14,18], sensor placement using only the measure of variance usually forces the sensors to be placed at the borders of the region under study, due to the fact that there is no measurement outside the region and, as a result, the borders tend to have very few measurements in their neighborhood to be used in the training set of GPs. To tackle this issue, in [16], a mutual information criterion is proposed in order to place the sensors at the locations most informative about the unseen locations. However, the optimization turns out to be an NP-hard problem. In [14], an approximate form of the mutual information optimization approach is considered to select informative sensor locations via exploiting the sub-modularity property of mutual information. However, if we continue to perform sensor placement successively using either the entropy of the region or the mutual information criterion, there is a high chance ending up with some sort of uniform sampling (equally spaced sensor placements) in the region. This is because the placements tend to occur at the locations far away from the visited locations of the sensors. These criteria only fulfill the exploration goals without taking into account refining measurements of the interesting features about the underlying phenomenon. In addition, the available budget on sensors may not allow us to have widely scattered sensor placements (or place the sensors far away from their previous positions) in applications such as the space missions that exemplify our work.

In this paper, we devise a general framework to specify the trajectory of mobile sensors in order to find and characterize the concentration of the quantity of interest for the PoI in the region under study. This work differs from previous work in this area because we seek to fulfill both exploration and data refinement desires. Here, we assume that there are some interesting phenomena occurring in the region under study. Therefore, the measurement of mutual information or entropy is not enough to reach a single objective. The proposed framework adaptively makes value-laden decisions on the sensor placement. This framework consists of two main stages. The first stage is the prediction stage, where we apply a Gaussian process regression model to predict the PoI at the unsampled locations. The GPR not only provides interpolated/extrapolated values but also the variance of the estimated values. Then, the question is how to decide on the next set of trajectories, based on the information obtained from the previous (GPR) stage. There is not sufficient information to obtain an optimal solution a priori, since the available information is local (the result of previous measurements) and may change over time. The decision between improving information in the neighborhood of known PoI and exploring new territory in the hope of discovering additional interesting locations can be hard or contradictory. In order to deal with the above issues, we set up the decision-making based on epistemic utility theory [36,37,38,39], which forms the second stage of our framework. Epistemic utility is able to provide decisions, called *satisficing* decisions, based on the local rather than global information in a way that specifically trades off between two different goals [40].

The remainder of this paper is organized as follows. In Section 2, some background of the GPR model is presented. Then, decision-making based on the epistemic utility controller is described. In Section 3, these tools are applied to the problem of trajectory determination of mobile sensors. Section 4 contains an example illustrating the effectiveness of the proposed approach.

## 2. Theoretical Preliminaries

This section is divided into two parts. The first part is devoted to a review on Gaussian process regression models, and the second part describes the epistemic utility theory. These two tools will serve as the backbone of our proposed framework.

### 2.1. Gaussian Process Regression

Gaussian processes (GPs) are widely used for modeling a feature or PoI based on observed spatiotemporal data. A GP can be used as a tool for either classification or regression, where the regression application of GP is called Gaussian process regression (GPR) [21,22]. It is assumed that the concentration of the physical quantity of interest can be evaluated via an unknown and probably nonlinear function, which we denote by f(·). The arguments of the function comprise a variable set u referred to as the input data. For example, u can be defined as $u\;=\;{\left[u_{x},u_{y},u_{z},t\right]}^{T}$, where $\left(u_{x},u_{y},u_{z}\right)$ and *t* denote the spatial and temporal information about the measurements, respectively. Unlike parametric models such as linear regression, GP is non-parametric. In GP, one defines a probability distribution as a prior over the unknown function f(·), directly. In other words, GP defines a distribution over functions in the function space and the inference is performed directly in this space [22]. This is more general than a parametric model such as Bayesian linear regression, where the prior distribution is defined over the space of parameters. The GP model treats any observation as an outcome of a Gaussian random variable, and all of these random variables are jointly Gaussian. With this setting, any well-defined GP model only needs a mean accompanied by a positive definite covariance function. Under this assumption, GP provides a posterior distribution over the unknown function *f* once data are observed. Therefore, for any set of *N* observations with the input data set $\left\{u_{1},\cdots ,u_{N}\right\}$, GP assumes that the distribution $p(f\left(u_{1}\right),\cdots ,f\left(u_{N}\right))$ is jointly Gaussian with some mean μ(U) and a covariance matrix K(U), where $U\;:=\;[u_{1},...,u_{N}]$. The entry in row *i* and column *j* of K(U) is denoted by ${\left[K\left(U\right)\right]}_{ij}\;=\;\kappa \left(u_{i},u_{j}\right)$, where κ(.,.) is a positive definite kernel function. The kernel function specifies the covariance between pairs of random variables at the corresponding data points. The GP model is defined as follows [41]:

(1) $$f\left(U\right)∼GP\left(\mu (U),K(U)\right),$$

where $f\left(U\right)\;:=\;{\left[f\left(u_{1}\right),\cdots ,f\left(u_{N}\right)\right]}^{T}$ and $\mu \left(U\right)\;:=\;{\left[\mu \left(u_{1}\right),\cdots ,\mu \left(u_{N}\right)\right]}^{T}$. For the regression purposes, GPR predicts the PoI at unseen data points using the available training data set. The GPR can handle noisy observations. Suppose we have access to a set of *N* noisy observations y=f(U)+ϵ, where $\epsilon \;∼\;N(0,\sigma_{n}^{2}I_{N})$ and $y_{n}\;=\;f\left(u_{n}\right)\;+\;\epsilon_{n},\forall n\;=\;1,\cdots ,N$. The pair $\left(u_{n},y_{n}\right)$ is the *n*th training data. Using GPR, the goal is to predict the underlying function *f* evaluated at some other input data set $U_{⋆}$ i.e., inferring $f(U_{⋆})$, where $U_{⋆}\;:=\;{\left[u_{⋆,1},\cdots ,u_{⋆,M}\right]}^{T}$. The set $U_{⋆}$ serves as the input test data set. Based on GP modeling, the prior joint distribution between the training and test data can be expressed as [41]

(2) $$\left[\begin{array}{c} y\\ f_{⋆} \end{array}\right]=N\left(\left[\begin{array}{c} \mu_{y}\\ \mu_{f_{⋆}} \end{array}\right],\left[\begin{array}{cc} K(U,U) & K(U_{⋆},U)\\ K(U,U_{⋆}) & K(U_{⋆},U_{⋆}) \end{array}\right]\right),$$

where $f_{⋆}$ denotes $f(U_{⋆})$ and ${\left[K\left(U,U_{⋆}\right)\right]}_{nm}\;=\;\kappa \left(u_{n},u_{⋆,m}\right)$. The predictive distribution over the test data, using the existing rules for conditioning Gaussian distributions, is expressed as follows:

(3) $$f_{⋆}|U,y,U_{⋆}∼N\left(\mu_{f_{⋆}},\Sigma_{f_{⋆}}\right),$$

where

(4) $$\begin{array}{cc} \mu_{f_{⋆}}\;= & \mu_{y}+K\left(U_{⋆},U\right){\left(K\left(U,U\right)+\sigma_{n}^{2}I\right)}^{-1}\left(y-\mu_{y}\right),\\ \Sigma_{f_{⋆}}\;= & K\left(U_{⋆},U_{⋆}\right)\;-\;K\left(U_{⋆},U\right){\left(K\left(U,U\right)\;+\;\sigma_{n}^{2}I\right)}^{-1}\;K\left(U,U_{⋆}\right). \end{array}$$

The point estimate for $f(U_{⋆})$ is the mean $\mu_{f_{⋆}}$ and the amount of uncertainty in the estimation is represented by the variance $\Sigma_{f_{⋆}}$ in Equation (4). Design of the covariance function requires incorporating some prior knowledge about the behavior of the PoI as it determines the amount of correlation between any pair of data points [21]. Some of the most widely used covariance functions are the squared exponential kernel ($\kappa_{SE}\left(u,u^{\prime }\right)=\exp\left\{-\frac{{\left(u-u^{\prime }\right)}^{2}}{2l^{2}}\right\}$) and rational quadratic ($\kappa_{RQ}\left(u,u^{\prime }\right)={\left(1+\frac{{\left(u-u^{\prime }\right)}^{2}}{2\alpha l^{2}}\right)}^{-\alpha }$), where *l* and α are hyperparameters [22]. These kernels fall in the category of stationary covariance functions. Once the structure of the covariance function is selected, the corresponding hyperparameters in the model can be chosen either empirically or using some quantified statistical methods. In the empirical approach, the selection of hyperparameters is usually achieved using the empirical features obtained from the observed data such as the smoothness or periodic behavior of the samples.

**Remark** **1.** *Although GPs are powerful tools for regression and classification problems, they suffer from high computational complexity as the sample size of the training data set increases. This problem occurs because the estimation of the test data involves inverting the covariance matrix of the training data which grows as more data are collected. The focus of this paper is not on the computational complexity of the GPs but rather on developing a general framework for mobile sensor configuration. Regarding the complexity of GPs, there exist some approaches such as the one for truncated covariance matrices in GPs [42], online sparse matrix GPs (OSMGP) algorithm [32], sparse greedy GP (SGGP) approximation method [43], and reduced rank GP (RRGP) [44]. One can use these approaches for the GPR estimation in the first stage of the proposed framework. Furthermore, there exist some studies on estimating the covariance matrix instead of an experimentally designed kernel function. For instance, Xu and Choi provide an approach to estimate and improve the quality of covariance function for anisotropic spatiotemporal GP using mobile sensor networks [13]. The suggested sampling method for such a problem is based on minimizing the information-theoretic cost function of the Fisher information [13]. This can also be incorporated into the proposed framework.*

### 2.2. Epistemic Utility Framework

When it comes to making a decision, the first approach choice that comes into mind is to seek only optimal decisions. However, it turns out that the demand for optimality may force us to modify the original optimization problem in an objectionable way in order to obtain a tractable computation. This demand may result in solving *another problem* in an optimal sense, which may not necessarily account for the original problem anymore [37]. A viable alternative to optimality seeking is to formulate *satisficing* decisions. A satisficing decision-making strategy looks for a satisfactory and sufficient solution for the original problem rather than seeking an optimal solution to a modified problem. This is useful when either the available information is local or insufficient, or the problem is complex enough that the optimal solution is intractable [37,40]. A simple example of this situation can be found in [37] for a routing problem, where a motorist wishes to reach a destination using a given map of city streets and information relating to the fuel costs of traversing each street under the assumption that the fuel costs are subject to change in arbitrary and unpredictable ways as time evolves. A satisficing approach operates like a rational, but not extremal-seeking, human driver would, finding a route that achieves the primary goal of arriving at the destination, reasonably take into account costs of travel, but without ensuring that a globally optimal solution is obtained.

Epistemic utility theory is a framework for making satisficing decisions. In epistemic utility theory, satisficing decisions are made via putting more emphasis on avoiding wrong decisions and favoring informationally valuable decisions rather than the more restrictive (and sometimes unachievable) task of finding the best solution. This theory employs two utility functions [37,39,45] and seeks a trade-off between them. This is different than a more conventional approach with a single utility for which a maximizing point is sought. The first utility function in the epistemic utility deals with the correctness of the decisions by characterizing the *truth value* of propositions being evaluated. The second utility function accounts for the importance of decisions by characterizing the *informational value* of rejecting propositions. The two utilities are established independently. As an example [40], the truth value for rival scientific theories can be assessed by compatibility with observations, while the informational value can be assessed by parsimony or predictive power.

To apply epistemic utility theory, each of these utility functions is equipped with the mathematical structure of a probability (through normalization if necessary), which allows these two utilities to be compared. Let $P=\{𝓅{\;}_{1},\cdots ,𝓅{\;}_{n}\}$ be a finite-dimensional decision space (propositions, or options available to an agent) and let F be the power set of P consisting of all subsets of P. Let q:F→[0,1] be a probability measure over the measurable space (P,F). The triple (P,F,q) defines a probability space, and the probability *q* can be interpreted as a measure of the truth value of the elements of F. The truth value utility function is also known as the *credal probability function* . Thus, for any set G∈F, q(G) is a measure of the truth value (credibility) of G [37]. Similarly, let m:F→[0,1] be another probability measure over the measurable space (P,F). The probability *m* is defined such that for any set G∈F, m(G) represents the informational value of rejecting G. The informational value utility function is also known as the rejectability probability function. The rejectability defines the amount of information the agent gains once some propositions are rejected from the decision space [36]. (If no options are rejected, the agent is left with the same uncertainty, so no information is gained.) After defining the credibility and rejectability probability functions, we can then formulate *satisficing decisions*. For this purpose, a parameter denoted as *agent’s index of boldness*, *b*, is used as a weighting factor on the informational value function. The boldness parameter represents the agent’s willingness, or boldness, to reject propositions. A larger boldness indicates a propensity to reject more options in the evaluation of the credal probability *q* vs. the informational probability *m*. Using Levi’s rule [45], among the set of possible options, those propositions whose truth value does not outweigh the weighted information value of rejection are rejected. That is, a proposition 𝓅 is not rejected if q(𝓅)>bm(𝓅). By this means, propositions are retained that are “good enough”, ensuring adequate performance without the imposition of an overly restrictive unique “optimal” solution.

For a decision space containing all the possible propositions P at some time, the set of options which are informationally valuable and have a probability of being correct is

(5) $$P_{l}=\left\{𝓅\;\in P:q\left(𝓅\;\right)\geq bm\left(𝓅\;\right)\right\}.$$

$P_{l}$ contains all the surviving, satisficing, propositions and thus it may not be a singleton set. The notion of satisficing here means that each element of $P_{l}$ is both likely to be correct and valuable in terms of gathering informational value. If the cardinality of the set $P_{l}$ is greater than 1, then the rejectability and credal probability functions of the surviving propositions are renormalized and the test in Equation (5) is repeated. This procedure is referred to as “deliberation”, and is repeated until the cardinality of $P_{l}$ cannot be reduced further [37]. We denote the surviving propositions of the deliberation stage as $P_{l}^{⋆}$. When there is still more than one decision acceptable and a single decision is needed, $P_{l}^{⋆}$ is then subjected to some “tie-breaking”. One way of doing this is decision rule [37]

(6) $${\hat{p}}_{{}_{B}}=\max_{𝓅\;\in P_{l^{⋆}}}\left\{q\left(𝓅\;\right)-bm\left(𝓅\;\right)\right\}.$$

## 3. Trajectory Determination of Mobile Sensors

In this section, we propose a general framework to determine the trajectory of mobile sensors in order to explore locations containing concentration of the physical quantity of interest in the region under study. Assume that an initial trajectory of the sensors has already been determined. As may happen in realistic situations, the measurements collected along these initial trajectories may not necessarily be very informative. Once some measurements are collected over the region, the goal becomes using the capability of sensors in follow-on trajectories to both refine the data and explore for interesting regions. Our proposed framework contains two main stages: the prediction stage and the decision stage. A single pass of the sensors only samples a small fraction of the entire region under study, so that decisions about whether to explore other unseen areas must be based on some predictions of the behavior of the interesting phenomenon. Such predictions will be conducive to decide on how to replace the mobile sensors or equivalently to determine the next set of trajectories of the existing sensors.

The prediction stage employs GPR modeling to estimate the PoI in unseen locations. In this setting, the collected data are treated as the training set, where the spatiotemporal location of the measurements determines the input training data and the corresponding measurements are the output training data. The input test data are the other unseen locations over the region under study and the output test data are unknown and are predicted using the GPR. Without loss of generality, we assume that input training data are collected into the set $U_{s}\in R^{N_{1}\times d}$, where $U_{s}=\left\{u_{1},\cdots ,u_{N_{1}}\right\}$ and *d* is the dimension of the input data i.e., $u_{n}\;\in \;R^{d}$. For example, d=4 for the spatiotemporal input data. The output training data, for the scalar output case, are accumulated in $y={\left[y_{1},\cdots ,y_{N_{1}}\right]}^{T}$, where $y_{n}$ is the output corresponding to the input $u_{n}$. The spatiotemporal information of the test data is collected into the set $U_{⋆}\in R^{N_{2}\times d}$, where $U_{⋆}=\left\{u_{⋆,1},\cdots ,u_{⋆,N_{2}}\right\}$. The unknown outputs evaluated at the input test data are defined as $f_{⋆}={\left[f\left(u_{⋆,1}\right),\cdots ,f\left(u_{⋆,N_{2}}\right)\right]}^{T}$. As prior knowledge, we assume that the joint density function between the training and test data is zero-mean Gaussian, meaning that on average we expect interesting phenomena to occur rarely. The GPR model was defined in Equation (2). The kernel function for constructing the covariance matrices K(·,·) in Equation (2) will be defined later. The predictive posterior distribution over $f_{⋆}$ for the noisy observation case was given in Equations (3) and (4).

**Figure 1:**
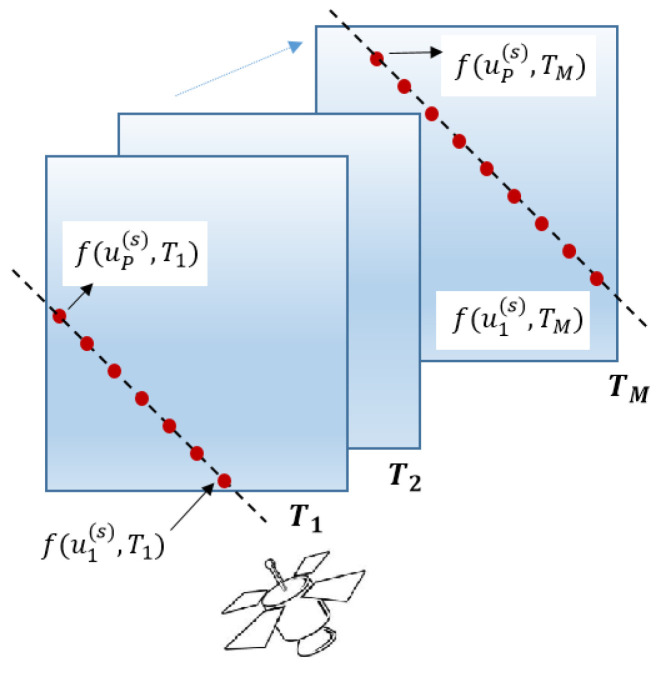
Example showing a satellite (or a mobile sensor) trajectories in the region under study.

In Figure 1, we show an example of sampling over the region under study.

In Figure 1, the dashed lines represent the trajectory of a satellite, the red circles denote the locations where the samples are taken, and the rectangular shape is the region under study. The function f(·) quantifies the physical PoI. The time frame $T_{m}$ is the *m*th time the satellite visits the region. Assume that the measurements during the time frame $T_{m}$ are taken much faster than the changes in the PoI. We also assume that the PoI exhibits some sort of smooth behavior in the region under study. Therefore, as the satellite is within a specific time frame $T_{m}$, we may expect to see high correlation between the nearby samples. We further assume (here, for simplicity) that the time it takes for the satellite to revisit the region is less than the time for changes to occur in the PoI. Therefore, if it happens to have the same trajectory for time frames $T_{i}$ and $T_{j}$, then a high correlation between the collected data at such time frames is expected for the case where $T_{i}$ is close to $T_{j}$.

Under the smoothness assumption for the PoI, and in order to account for the correlation that may exist between the nearby measurements, we define the following squared exponential covariance function

(7) $$\kappa \left(u_{i},u_{j}\right)=\exp\left\{\frac{-∥u_{i}-u_{j}{∥}_{2}^{2}}{2l_{1}}+\frac{-∥T_{m_{1}}-T_{m_{2}}{∥}_{2}^{2}}{2l_{2}}\right\},$$

where $l_{1}$ and $l_{2}$ are scaling factors, $u_{i}$ and $u_{j}$ are the spatial information, coordinates, of any two arbitrary locations in the region under study. The terms $T_{m_{1}}$ and $T_{m_{2}}$ are the time frames corresponding to the temporal information related to $u_{i}$ and $u_{j}$, respectively. As the available prior knowledge changes, one may define a different kernel function from Equation (7).

Suppose that $S=\{s^{\left(1\right)},s^{\left(2\right)},\cdots ,s^{\left(K\right)}\}$ is a set of all feasible trajectories of the sensors through the region under study. We denote the locations along the trajectory $s^{\left(k\right)}$ at which measurements are collected by the set $U^{s^{\left(k\right)}}=\left\{u_{1}^{s^{\left(k\right)}},\cdots ,u_{P}^{s^{\left(k\right)}}\right\}$. For the case where the trajectory along $s^{\left(k\right)}$ has not been taken yet, the locations in the set $U^{s^{\left(k\right)}}$ belong to the set of test data i.e., $U^{s^{\left(k\right)}}\in U_{⋆}$.

Using the GPR model, we obtain a prediction of the PoI throughout the region and the amount of uncertainty (variance) associated with the predictions. The amount of uncertainty can be evaluated via the kernel function defined in Equation (7). We denote $\hat{f}\left(U_{p}^{s^{\left(k\right)}}\right)$ and $\hat{\Sigma }\left(U_{p}^{s^{\left(k\right)}}\right)$ as the estimate quantifying the PoI and the associated measure of variance for the *p*th location along the trajectory $s^{\left(k\right)}$, respectively. In this setting, we assume that the locations containing a high concentration of the physical PoI have a higher value of the quantifying function f(·) compared to the other locations. Then, the goal is to decide on the next trajectory of the sensors based on the available data, the estimated data, and also the amount of uncertainty over the region in order to explore the interesting phenomenon. Although the GPR provides us with an estimate of the PoI over the whole region, we get large uncertainty at locations where there exist almost no measurements; the farther away the sensors are from the measurements, the higher the variance becomes. Therefore, if we only emphasize refining existing measurements, the sensors lose the inclination to explore. In contrast, if we put more emphasis on the variance, then the sensors are more encouraged to choose the trajectories which are far away from the previous trajectories to fulfill the exploration objective of the mission. In this case, even if interesting phenomena are found by the past trajectories, the sensors are reluctant to pass nearby again. One way of taking into account both data refinement and exploration desires can be achieved by constructing the following optimization problem to decide on the trajectories of the sensors:

(8) $$k^{⋆}=\underset{k}{\arg\;\max}\;{\hat{f}}_{ave}\left(U^{s^{\left(k\right)}}\right)+\lambda {\hat{\Sigma }}_{ave}\left(U^{s^{\left(k\right)}}\right),$$

where *k* denotes the *k*th trajectory from the dictionary of feasible trajectories S that pass through the region under study. The parameter λ is a tuning parameter that balances between the desire to explore and refine data in regions known or predicted to be of interest. Notice that, since the set S is defined in such a way to only cover the region under study, the maximization problem for small λ will at most result in the selection of a trajectory at the boundary of the region. In Equation (8), ${\hat{f}}_{ave}\left(U^{s^{\left(k\right)}}\right)$ and ${\hat{\Sigma }}_{ave}\left(U^{s^{\left(k\right)}}\right)$ are defined as

(9) $$\begin{array}{c} {\hat{f}}_{ave}\left(U^{s^{\left(k\right)}}\right)=\frac{1}{P}\sum_{p=1}^{P}\hat{f}\left(u_{p}^{s^{\left(k\right)}}\right),\\ {\hat{\Sigma }}_{ave}\left(U^{s^{\left(k\right)}}\right)=\frac{1}{P}\sum_{p=1}^{P}\hat{\Sigma }\left(u_{p}^{s^{\left(k\right)}}\right). \end{array}$$

The optimization problem Equation (8) results in a single solution, which represents an optimum (of a λ-weighted utility function). Notice that there exist no globally optimal solution for the optimization problem in Equation (8) based on the relatively small amount of information that can be obtained from the region under study, the tension that exists between the two desiderata of exploration and data refinement in the region, and the locality of the information that we obtain. The epistemic utility framework is well-suited to handle these types of problems by making satisficing decisions such that the selected trajectories are informationally valuable decisions. Application of epistemic utility is accomplished by defining the two probability functions so that the decision-maker meets both goals of exploration and data refinement. In this setting, each of the possible trajectories of sensors is considered as a hypothesis. In contrast to the optimization problem in Equation (8), the decision maker with Equation (5) uses the credibility and rejectability probability functions which is a comparison between these two probability functions with the emphasizing factor *b*, where *b* denotes how much emphasis we consider for the informational value that we can obtain by rejecting propositions (trajectories) from the decision space. The boldness factor *b* in Equation (5) represents the agent’s willingness to reject propositions, which makes it totally different than the term λ in Equation (8). For b∈[0,1], the higher value of *b* results in more rejection of possible propositions (decisions). For example, setting b=1 corresponds to rejecting as many propositions as possible in the decision space.

Based on the discussion provided above, below we formulate the problem using the epistemic utility framework represented in Section 2.2. Specifically, we consider the two following cases with their corresponding credal and rejection probability functions. In the first case, we define the informationally valuable trajectories as the trajectories that pass through the regions with high entropy (that is, passing through those regions will resolve a high uncertainty). Correspondingly, we emphasize the rejection of those trajectories which pass through regions with low entropy. Therefore, the rejectability probability is defined to emphasize rejecting the trajectories from S that contain lower uncertainty in order to favor the desire for exploration. Since the kernel function defined in Equation (7) becomes small when the desired trajectory $S^{\left(k\right)}$ is far from previous trajectories, the uncertainty associated with this trajectory, as defined in Equation (9), will be larger. This can be seen in Equation (2) when the covariance $K(U,U_{⋆})$ would be small and $\Sigma_{f_{⋆}}$ large as a result. The probability functions constructing the epistemic utility controller can be written as

(10) $$\begin{array}{c} q\left(U^{s^{\left(k\right)}}\right)=\hat{f_{n}}\left(U^{s^{\left(k\right)}}\right),\;\forall k=1,2,...,K,\\ m\left(U^{s^{\left(k\right)}}\right)=\frac{1}{{\hat{\Sigma }}_{n}\left(U^{s^{\left(k\right)}}\right)},\;\forall k=1,2,...,K, \end{array}$$

where ${\hat{f}}_{n}\left(U^{s^{\left(k\right)}}\right)$ and ${\hat{\Sigma }}_{n}\left(U^{s^{\left(k\right)}}\right)$ are defined as

(11) $$\begin{array}{c} {\hat{f}}_{n}\left(U^{s^{\left(k\right)}}\right)=\frac{{\hat{f}}_{ave}\left(U^{s^{\left(k\right)}}\right)}{\sum_{k=1}^{K}{\hat{f}}_{ave}\left(U^{s^{\left(k\right)}}\right)},\;\forall k=1,2,...,K,\\ {\hat{\Sigma }}_{n}\left(U^{s^{\left(k\right)}}\right)=\frac{{\hat{\Sigma }}_{ave}\left(U^{s^{\left(k\right)}}\right)}{\sum_{k=1}^{K}{\hat{\Sigma }}_{ave}\left(U^{s^{\left(k\right)}}\right)},\;\forall k=1,2,...,K, \end{array}$$

and where ${\hat{f}}_{ave}\left(U^{s^{\left(k\right)}}\right)$ and ${\hat{\Sigma }}_{ave}\left(U^{s^{\left(k\right)}}\right)$ were defined in Equation (9). The subscript *n* denotes that the functions in Equation (11) are normalized to act like probabilities. According to Equation (10), we assign credal probabilities to the estimates of each possible trajectory while the measure of uncertainties determine the rejection probabilities. Without loss of generality, we assume that the more interesting behavior the phenomenon at location $u_{p}^{s^{\left(k\right)}}$ becomes, the higher value the underlying function $\hat{f}\left(u_{p}^{s^{\left(k\right)}}\right)$ possesses.

In the second case, we relate the less interesting phenomenon (low concentration of the quantity of interest) to the rejection probability function. Particularly, the less interesting the predicted phenomenon behaves along the possible trajectory $U^{s^{\left(k\right)}}$, the higher the rejection of the corresponding hypothesis becomes. The credal probability is evaluated via the amount of uncertainty each possible trajectory may possess. The credal and rejection probabilities for case 2 are

(12) $$\begin{array}{c} q\left(U^{s^{\left(k\right)}}\right)={\hat{\Sigma }}_{n}\left(U^{s^{\left(k\right)}}\right),\;\forall k=1,2,...,K,\\ m\left(U^{s^{\left(k\right)}}\right)=\frac{1}{\hat{f_{n}}\left(U^{s^{\left(k\right)}}\right)},\;\forall k=1,2,...,K, \end{array}$$

where ${\hat{f}}_{n}\left(U^{s^{\left(k\right)}}\right)$ and ${\hat{\Sigma }}_{n}\left(U^{s^{\left(k\right)}}\right)$ were defined in Equation (11). Once the probability functions are computed for either of the two above cases, we apply Levi’s rule of epistemic utility. As a result, only those trajectories that satisfy $q\left(U^{s^{\left(k\right)}}\right)\geq bm\left(U^{s^{\left(k\right)}}\right)$ are surviving hypotheses. The surviving options are defined by the following set:

(13) $$U_{srv}=\left\{u\in U_{⋆}\;:\;q\left(u\right)\geq bm\left(u\right)\right\}.$$

**Remark** **2.** *In Equations (10) and (12), we used the normalized version of estimates and the associated variance along each trajectory in order to make q(·) and m(·) follow the “sum to one” property of probability. However, one can remove the normalization step and define the boldness factor of b≥0 instead of b∈[0,1].*

**Figure 2:**
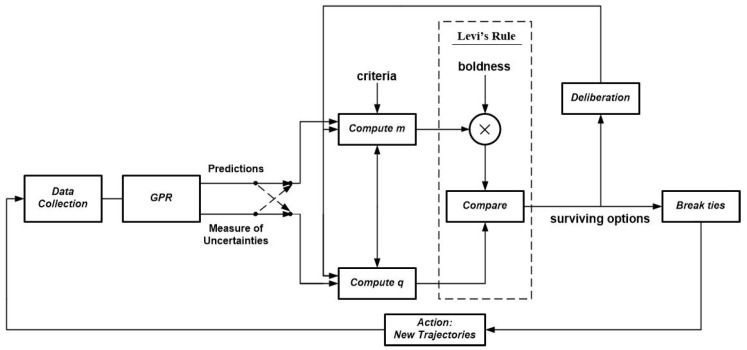
Block diagram of exploration using mobile sensors based on epistemic utility controller.

After applying the rule defined in Equation (13), the number of surviving options may not be necessarily unique i.e., the cardinality of $U_{srv}$ could be greater than one. Each of the elements of $U_{srv}$ is a satisficing hypothesis meaning that it is both likely to be correct and possess high informational value. In order to take action, we seek to accept only one trajectory (one hypothesis). Reducing the number of elements in the set $U_{srv}$ is accomplished in the deliberation stage described in Section 2.2 results in reducing the number of surviving hypotheses. Once the cardinality of the set $U_{srv}$ reduces to a reasonable number, the tie-breaking stage comes into play to force the set $U_{srv}$ to a unique element in order to take an action. For this purpose, one can apply the approach with Equation (6) as described in [37], which selects one hypothesis out of the survived hypotheses as the next trajectory. We refer to this trajectory as $U^{s^{\left(k_{⋆}\right)}}$. Finally, after measurement, the data obtained from $U^{s^{\left(k_{⋆}\right)}}$ are added to the training set *U*, and the whole process starts again. Figure 2 illustrates the block diagram of the proposed framework.

In order to restrain the increase in the amount of training data fed to the GPR (to reduce complexity), in the data collection block of Figure 2, we only retain the measurements obtained from the last *M* visited trajectories of sensors. Once a new trajectory is determined and the corresponding measurements are collected, the new information is added to the training set and the oldest set of measurements are discarded. The reason is due to the assumption that the oldest set of measurements may have a very low correlation with the new measurements and the PoI may have been changed. This avoids dealing with the inverse of a big covariance matrix of the training data as we continue collecting new measurements.

## 4. Simulation Results

**Figure 3:**
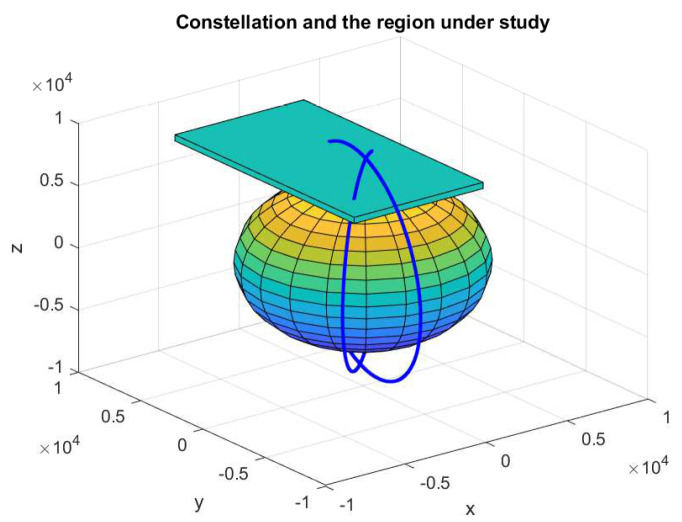
The satellites orbits and the region under study.

We demonstrate how the proposed framework is applied via a surrogate problem using a constellation of two satellites at the low Earth orbit (LEO). In this particular problem, we are incapable of performing random sampling over the region. Instead, we are restricted to follow specific trajectories once the orbits of the satellites (or trajectories of the mobile sensors) are determined. Initially, the satellites move in predetermined orbital planes over the region under study. For simplicity, assume that the PoI remains unchanged during the sampling period. Figure 3 illustrates an example including the orbital planes of satellites, where the rectangular slab indicates the region under study.

**Figure 4:**
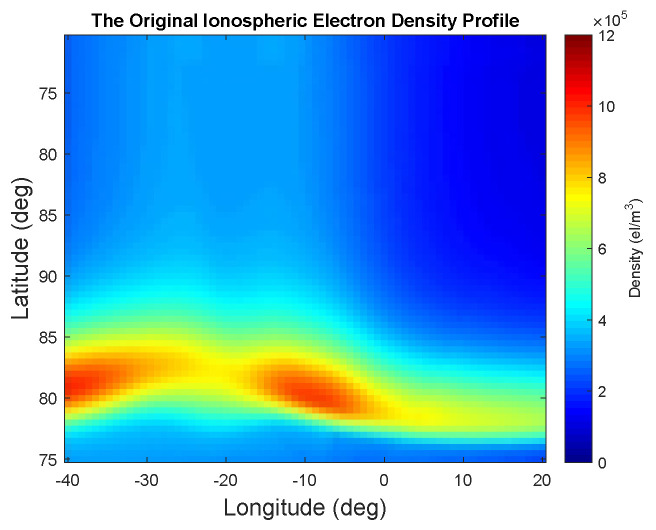
Simulated ionospheric electron density profile, representing the PoI.

In Figure 3, the constellation is defined based on Keplerian orbital elements with semi-major axis a={7700,8500} (km), eccentricity e=0, inclination of i={π/2,π/2} (rad), and the right ascension of ascending node (R.A.A.N.) Ω={π/4,2π/5} (rad). Since the PoI is assumed to be unchanged during the sampling period, the fastest varying orbital elements, the true (mean) anomaly θ and the argument of perigee are ignored. However, one can also take these two orbital elements into account for the case when the PoI changes over the sampling period. The region under study shown in Figure 3 corresponds to the Earth coverage with latitude range of [72.74°, 90°], longitude range of [−37.84°, 19.27°], and for the altitude range of [129 Km, 629 Km]. The PoI that we consider here is a simulated measure of total electron content (TEC) in the ionosphere. The profile of TEC is illustrated in Figure 4, which is borrowed from [46].

In Figure 4, the highest and the lowest quantity of the interesting phenomenon corresponding to the PoI, the highest and lowest quantities of the electron density, are shown with red and blue colors, respectively. We further assume that the PoI has the same profile for the altitude range under study ([129 Km, 629 Km]) as shown in Figure 4 along the *z*-axis of the rectangular region shown in Figure 3. This image can be thought of as a discretized 2D version of the region of interest defined by the pixel values. In the simulations, it is assumed that it is possible to get direct measurements about the PoI along the current trajectories of satellites. Since the electron density content profile, as the PoI for our case study, is assumed to be unchanged during the sampling period, the kernel function in Equation (7) simplifies into

(14) $$\kappa \left(u_{i},u_{j}\right)=\exp\left\{\frac{-∥u_{i}-u_{j}{)∥}_{2}}{2l}\right\},$$

where we set l=10 and $u_{i}$ is defined by the pixel location of the image shown in Figure 4.

**Figure 5:**
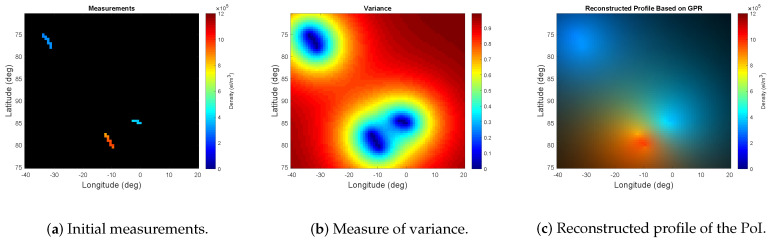
From (**a**–**c**): Results of the initial measurements, measure of variance, and the reconstructed profile of the PoI based on the initial observation and applying the first stage of the proposed framework.

From the initial trajectories, shown in Figure 3, the corresponding set of measurements of the region under study is shown in Figure 5a. From the initial measurements, the GPR model defined in Equation (4) and Equation (14) is applied to measure the uncertainty and the estimate of the TEC throughout the region under study. The results are illustrated in Figure 5b,c.

The second stage of the proposed framework is applied to decide on the next trajectories of the satellites. Although the initial orbital planes were constructed directly from the Keplerian orbital planes, below we assume (for simplicity in this example) that each possible trajectory can pass through the region under study and measure the TEC with the coverage Earth’s longitude resolution of 0.5° as shown in Figure 4. The problem of designing the Keplerian orbital elements to generate the actual orbits corresponding to such trajectories is not the focus of this paper and is considered as a future work.

**Figure 6:**
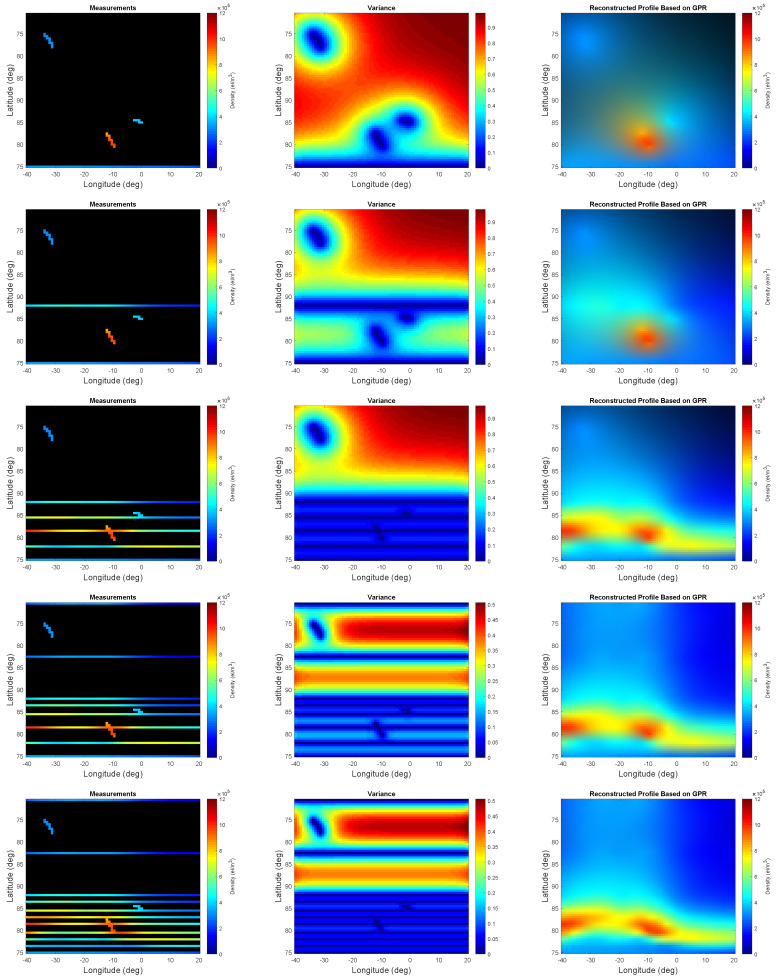
From (**left**) to (**right**), the measurements, measure of uncertainty, and the reconstructed profile of the phenomenon based on the initial and one, two, five, eight, and eleven successive trajectories are shown for b=1 when using Equation (12).

The epistemic utility is set with the agent’s index of boldness b=1 and the credal and rejection probability functions in Equation (12). In other words, the rejection probability function is defined such that it tends to remove the trajectories corresponding to regions with low electron density content. The credal probability function is set to encourage trajectories willing to visit regions with higher uncertainty. Figure 6 illustrates some of the results for the measurements corresponding to the selected trajectories, measure of uncertainty, and the reconstruction profile.

According to Figure 6, the selected trajectories are not willing to revisit the vicinity of the areas which seem to contain low electron density. Simultaneously, the decision maker does not allow for accepting a trajectory at the very vicinity of the already chosen trajectories even if high electron density has been detected at their neighborhood. This is shown by the measure of variance in the third row of Figure 6. More specifically, very few trajectories are selected at the subregions with low electron density content, and such trajectories demonstrate some sort of uniform sampling. In contrast, more trajectories are chosen in the interesting subregions and yet these trajectories do not tend to be next to each other.

**Figure 7:**
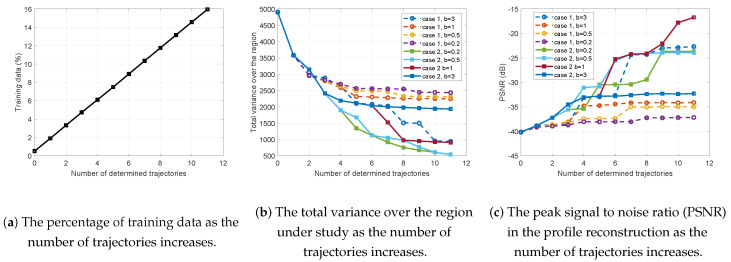
Comparison of the performance for cases 1 and 2 with b=0.2,0.5,1, and b=3.

In Figure 7, we compare the performance of the proposed framework for this example for cases 1 and 2 defined in Equation (10) and Equation (12), respectively. In Figure 7a, we show the percentage of the measurements (training data) with respect to the total number of possible data if we were able to cover all the regions under study. Even after accumulating the measurements obtained from the initial and the eleven successive trajectories, we still cover around 16% of the complete data over the region. In Figure 7b, the total variance over the region vs. the increase in the number of trajectories is illustrated. Here, the variance of visited locations are set to zero and the variance of unvisited locations are measured via the kernel function defined in Equation (14). Then, we sum over all the variances associated with the pixels. Finally, in Figure 7c, the peak-SNR evaluation between the true and the reconstructed TEC profile is demonstrated.

According to Figure 7b, the increase of boldness factor for case 1 results in the decrease of the overall uncertainty in the region under study. This is because the rejection probability function corresponds to the inverse of overall variance along the possible trajectories. Therefore, the increase of the boldness factor promotes selecting the trajectories along which higher uncertainty is predicted. In contrast, case 2 shows a different behavior in which the increase of the boldness factor forces discarding the trajectories that are believed to result in collecting measurements in the subregions with low TEC. The peak signal to noise ratio (PSNR) evaluation depends not only on how to construct the probabilities in the epistemic utility but also on the true behavior of the PoI. Since the PoI has a smooth behavior and we have already taken this fact into account, the PSNR increases as we increase the boldness factor for case 1. For case 2, the reduction of the boldness factor usually provides better performance in terms of PSNR, but it also depends on where we sample. For example, setting b=0.2 does not show better performance compared to b=0.5 and b=1. The reason is that the controller decided on a trajectory that is in the region with high TEC but close to the edge of such a phenomenon. Since the kernel function in the GPR stage assumes the smooth behavior, it expands the interesting phenomenon at the neighborhood of the selected trajectory and thus the estimated PoI exceeds the edges of the true PoI. This yields to a decrease in the PSNR.

**Figure 8:**
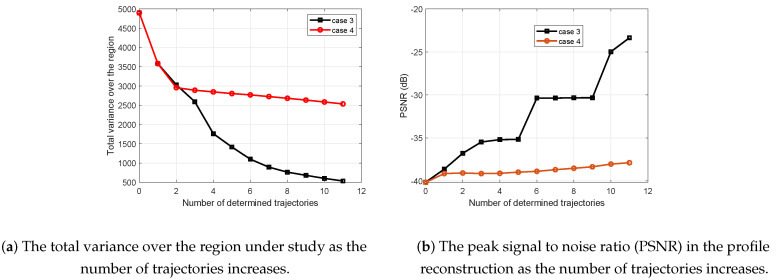
Comparison of the performance for cases 3 and 4.

**Figure 9:**
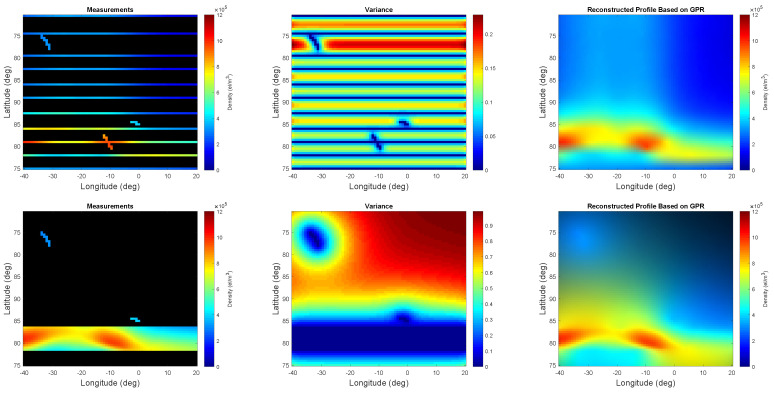
From (**left**) to (**right**), the measurements, measure of uncertainty, and the reconstructed profile of the phenomenon based on the initial and eleven successive trajectories are shown for case 3 (first row) and case 4 (second row), respectively.

Finally, we consider two more cases to highlight the advantage of the proposed framework. In cases 3 and 4, the trajectories are selected only based on either the variance or the quantity of the interesting phenomenon, respectively. Notice that case 3 tends to select the trajectories with highest entropy (variance). Using the variance for sensor placement has been widely used [17,18,47], where we have modified it such that it can be used for making decisions on the trajectories of mobile sensors rather than the pointwise sensor placement problems. In fact, case 3 and case 4 correspond to solving the optimization problem in Equation (8) when the terms ${\hat{f}}_{ave}\left(U^{s^{\left(k\right)}}\right)$ and ${\hat{\Sigma }}_{ave}\left(U^{s^{\left(k\right)}}\right)$ are discarded, respectively. Figure 8 and Figure 9 illustrate the obtained results.

As expected, Figure 8 shows that case 3 outperforms case 4 in terms of reducing the overall uncertainty in the region. The reason is that case 3 only favors the trajectories with the highest variance. In addition, due to the smoothness of the PoI, case 3 still shows higher PSNR than case 4. Comparing the total variance and the PSNR of all cases 1–4, it is clear that cases 1 and 2 result in better performance in reducing the overall variance and the increase of PSNR, collectively. The trajectory selection of cases 3 and 4 is also shown in Figure 9 for eleven successive sets of measurements added to the initial measurements. Comparing cases 3 and 4, it is obvious that they both tend to uniformly sample the region. However, the difference is that case 3 uniformly samples the whole region, while case 4 uniformly samples the vicinity of the interesting phenomenon once some are observed. Therefore, if there was any other interesting subregion, we would not have a chance to observe it via case 4 for a low number of trajectories. In contrast, cases 1 and 2 of our proposed framework apply an adaptive sampling structure to balance between the reduction of the amount of uncertainty in the region and refining data in the vicinity of an interesting phenomenon.

**Remark** **3.** *For the purpose of only showing how the principles of the proposed framework can be applied, the simulations were carried out by neglecting the constraint on the ΔV-budget of the satellites for the orbit transfer to reach the trajectories selected by the decision-maker. Thus, in a realistic case scenario, some of the selected trajectories may not be feasible. However, this issue can be resolved by discarding non-affordable trajectories from the dictionary of trajectories in the region under study and then applying Equation (13).*

## 5. Conclusions

We developed a new framework for placing mobile sensors using Gaussian process regression and the epistemic utility controller. The proposed framework considers both desires of exploration and data refinement once some interesting phenomenon is observed. This approach is useful for problems where very little local information is available and no optimal solution exists for the sensor placement. The proposed framework is constructed in a general way and, with some further modifications, it can also handle the constraints that may exist on the budget for sensor placement.

## 6. Future Work

As a future work, we will take into account a more realistic set of constraints that may exist in the decision-maker stage of the proposed framework. For example, some common constraints on the constellation design problem are on the feasibility of the orbital planes and the available ΔV-budget of the satellites, which leads to some modifications in Equation (13) of the decision maker stage and the way the feasible trajectory dictionary S is constructed. We will also investigate the comparison of the epistemic utility-based decisions with other techniques such as reinforcement learning.
